# The chemokine receptor CXCR4 and its ligand CXCL12 are activated during implantation and placentation in sheep

**DOI:** 10.1186/1477-7827-9-148

**Published:** 2011-11-03

**Authors:** Ryan L Ashley, Alfredo Q Antoniazzi, Russell V Anthony, Thomas R Hansen

**Affiliations:** 1 Department of Animal and Range Sciences, New Mexico State University, Las Cruces, New Mexico, USA; 2 Department of Biomedical Sciences, Animal Reproduction and Biotechnology Laboratory, Colorado State University, Fort Collins, Colorado, USA

**Keywords:** CXCR4, CXCL12, Placenta, Trophoblast, Implantation, Sheep

## Abstract

**Background:**

The progression of implantation and placentation in ruminants is complex and is regulated by interplay between sex steroids and local signaling molecules, many of which have immune function. Chemokines and their receptors are pivotal factors in implantation and vascularization of the placenta. Based on known critical roles for chemokine receptor 4 (CXCR4) during early pregnancy in other species, we hypothesized that CXCR4 and its ligand CXCL12 would increase in the endometrium and conceptus in response to implantation in ewes. The objectives of the current study were to determine if CXCL12 and CXCR4 were upregulated in: endometrium from pregnant compared to non-pregnant ewes and in, conceptuses, cotyledons, caruncles and intercaruncular tissue.

**Methods:**

Tissues were collected from sheep on Days 12, 13, 14, and 15 of either the estrous cycle or pregnancy and from pregnant ewes on Days 35 and 50. Blood samples from jugular and uterine vein were also collected on all days. Conceptuses were collected from mature ewes on Days 13, 15, 16, 17, 21 and 30 of gestation. Real time PCR was used to determine relative mRNA concentrations for CXCL12 and CXCR4 and Western blot analysis was employed to confirm protein concentration.

**Results:**

Differences described are P < 0.05. In the endometrium, CXCR4 mRNA and protein was greater on Day 15 of pregnancy compared to the estrous cycle. CXCL12 and CXCR4 mRNA in conceptuses was greater on Days 21 and 30 compared to earlier days. CXCL12 mRNA was greater in cotyledons on Day 35 compared to Day 50. On Day 35 of gestation, CXCR4 was greater compared to Day 50 in caruncle and intercaruncular tissue. White blood cells obtained from jugular and uterine vein collection had the greatest mRNA concentration of CXCL12 on Day 35 of pregnancy.

**Conclusions:**

A comprehensive analysis of CXCL12 and CXCR4 expression in fetal and maternal tissues during early pregnancy is reported with noteworthy differences occurring during implantation and placentation in sheep. We interpreted these data to mean that the CXCL12/CXCR4 pathway is activated during implantation and placentation in sheep and is likely playing a role in the communication between trophoblast cells and the maternal endometrium.

## Background

The immunological mechanisms that govern success of pregnancy are multifaceted. A remarkable attribute of normal pregnancy is the delicate communication that exists between trophoblast cells and differentiated maternal cells in the uterus. These cells communicate via production of cytokines, chemokines, growth factors and hormones to establish a unique maternal-fetal immune environment that contributes to fetal survival and "programming" of the maternal uterus until parturition [1-5]. Interest in chemokines and their receptors during implantation has grown tremendously in the last two decades because the endometrial epithelium produces and secretes chemokines [6,7]. During early gestation, leukocytes are recruited into the endometrium and regulation of uterine tissue is thought to be orchestrated by an array of chemokines in a precise spatial and temporal pattern [8,9].

The progression of implantation and placentation in ruminants is a complex and prolonged process that is regulated by interplay between sex steroids and local signaling molecules, many of which have immune function. Chemokines and their receptors are pivotal factors in implantation and vascularization of the placenta. Chemokine receptor 4 (CXCR4), is specifically up regulated in human endometrium during the implantation window [10] and increased immunostaining for CXCR4 is observed in cultured endometrial epithelium only when a blastocyst is present [10,11]. Additionally, varying temporal expression of CXCR4 in human placenta exists, with much greater CXCR4 expression observed in early compared to term placenta, suggestive of an important role for CXCR4 during early placental development [12]. CXCR4 is expressed in a multitude of tissues and cell types, including neutrophils, all B cells and monocytes, the majority of T-lymphocytes, endothelial cells, and epithelial cells [13] and its primary ligand is the stromal derived factor-1, also known as CXCL12.

The role of CXCR4 is also implicated in cross talk between trophoblasts and endometrium by recruiting lymphocytes into decidua and stimulating trophoblast proliferation and invasion [14-16]. Treatment of trophoblast cells with recombinant CXCL12 results in increased viability and activation of MAPK ERK1/2 pathway suggesting that CXCL12/CXCR4 interactions play an important role in early pregnancy in humans [16].

In humans, similar to other mammals, most of the angiogenesis in the embryo and placenta occurs in the first trimester, which correlates with intense expression of CXCR4 in placental tissue from early pregnancies [12]. It is not surprising that CXCR4 may affect angiogenesis, as the chemokine system is a major regulator of angiogenesis as reviewed by Rosenkilde and Schwartz [17].

A detailed analysis of CXCL12 and CXCR4 in the ovine endometrium, conceptus, and placental tissue during early gestation has not been reported. Based on known critical roles of CXCR4 during early pregnancy in other species, we hypothesized that expression of CXCL12 and CXCR4 in the endometrium and conceptus increases during the time of implantation and placentation in ewes to facilitate communication between trophoblast cells and maternal endometrium. The objectives of the current study were to determine if CXCL12 and CXCR4 was differentially expressed in: endometrium from pregnant and non-pregnant ewes on Days 12, 13, 14 and 15; ovine conceptuses on Days 13, 15, 16, 17, 21 and 30 after mating; and cotyledons, caruncles and intercaruncular tissue from ewes on Days 35 and 50 of gestation.

## Methods

### Animals

All experimental procedures using animals were reviewed and approved by the Colorado State University Animal Care and Use Committee. Estrus was synchronized in mature, white-faced, western range ewes during the mid to late luteal phase with two injections of dinoprost tromethamine (5 mg i.m.; Lutalyse; Pfizer, New York, NY) administered 4 h apart. Upon detection of estrus (Day 0) with a caudaepididyectomized ram, ewes were placed into experimental groups.

For studies comparing estrous cycle versus pregnancy, ewes (n = 5 to 6 ewes per day) were mated to an intact or caudaepididyectomized ram and then anesthetized with sodium pentobarbitol (20 mg/kg, i.v.) on either Day 12, 13, 14, or 15 of the estrous cycle, or Day 12, 13, 14, 15, 35 or 50 of pregnancy. Prior to anesthetization, jugular venous blood samples were collected for plasma from each ewe. A midventral laparotomy was performed to expose the reproductive tract, and uterine venous blood was collected for plasma. The reproductive tract was removed, tissues were collected and snap frozen in liquid nitrogen and stored at -80°C for subsequent RNA and protein extraction and the ewes were euthanized by exsanguination. Pregnancy was confirmed by the presence of a conceptus. Blood samples were centrifuged (30 min, 2500 × g, 4°C), white blood cells were removed and placed in Tri Reagent BD (Molecular Research Center, Inc. Cincinnati, OH) and then stored at -80°C for RNA isolation.

Conceptuses were collected from mature crossbred ewes on Days 13, 15, 16, 17, 21, and 30 of gestation (n = 4 to 6 conceptuses per day), as previously described by Purcell and colleagues [18]. Briefly, for collection of Day 13 conceptus tissues, ewes were superovulated by twice-daily i.m. injections for 4 days of follicle-stimulating hormone (FSH; 30, 30, 20, 20, 10, 10, 10, and 10 mg of Folltropin; Bioniche, Belleville, ON, Canada) beginning on Day 7 of the estrous cycle. Dinoprost tromethamine (Lutalyse; Pfizer, New York, NY) was administered in two doses (5 mg, i.m., per dose) given 4 h apart beginning at the time of the sixth FSH injection. Conceptuses on Days 15 through 30 of gestation were collected from ewes synchronized by two injections (10 mg, i.m.) of Lutalyse given 14 days apart. After observation of standing estrus with a vasectomized ram, all ewes were bred by one of two intact rams. Pregnant ewes were sedated using sodium pentobarbital (20 mg/kg, i.v.), a complete hysterectomy was performed, and the ewes were euthanized (90 mg/kg sodium pentobarbital, i.v.). Conceptuses were flushed from the uterus using Dulbecco modified Eagle medium/Nutrient Mixture F-12 Ham (DMEM/F12; Sigma, St. Louis, MO) supplemented with 0.25% Fraction V BSA (Sigma) warmed to 38°C. All recovered conceptuses were placed into 1.5-ml Eppendorf tubes and briefly centrifuged before removal of any liquid, and then they were frozen at -80°C until RNA isolation. For Day 21 and Day 30 conceptuses, the fetus was separated from the trophoblast before freezing.

### RNA and protein isolation

Total RNA was isolated from sheep tissues (not conceptus) using Tri Reagent (Sigma Chemical Co., St. Louis, MO). Each RNA sample was subjected to RNase-free DNase treatment (QIAGEN, Valencia, CA) to eliminate genomic DNA contamination and further purified using the RNeasy Mini Elute clean up kit (QIAGEN, Valencia, CA). The quantity and purity of RNA were determined using a NanoDrop ND-1000 Spectrophotometer. For conceptuses, total cellular RNA was isolated from individual conceptuses using RNeasy kits (Qiagen, Valencia, CA) according to the manufacturer's protocol. Quantity and integrity of RNA were confirmed by the absorbance ratio at 260:280 nm and by electrophoresis on a 1.2% formaldehyde denaturing gel and visualization of the 18S and 28S ribosomal subunits. RNA samples were stored at -80°C until use.

Protein was isolated from ovine tissues (100 mg/sample) by homogenizing in 1 mL of lysis buffer (20 mM Tris (pH 8.0), 137 mM NaCl, 10% glycerol, 1% Nonidet P-40, 0.1% sodium dodecyl sulfate SDS, 0.5% deoxycholate, and 0.2 mM phenylmethylsulfonyl fluoride) supplemented with protease inhibitor cocktail (Roche, Applied Science, Indianapolis, IN). Samples were placed on ice for 15 min and then centrifuged at 10,000 × g for 10 min at 4°C and supernatants subsequently removed and stored at -80°C. Concentrations of protein were determined using BCA protein assay (Pierce, Rockford, IL).

### Real-time PCR (qPCR)

Synthesis of cDNA was accomplished by using the iScript cDNA Synthesis Kit employing the reverse transcriptase (RT) RNAse H+ (Bio-Rad Laboratories, Hercules, CA) per manufacturer's instructions. The RT products were diluted to a final volume of 100 μL. Real-time PCR (qPCR) was performed using a LightCycler 480 PCR Detection System (Roche Applied Science, Indianapolis, IN) and components of the iQ SYBR Green supermix (Bio-Rad Laboratories, Hercules, CA), 0.525 μM forward and reverse specific primers, and 2 μL of cDNA. The specific primers employed included: GAPDH forward primer, 5'-TGACCCCTTCATTGACCTTC-3', GAPDH reverse primer, 5'-CGTTCTCTGCCTTGACTGTG-3', CXCL12 forward primer, 5'-CCTTGCCGATTCTTTGAGAG-3', CXCL12 reverse primer, 5'-GGTCAATGCACACTTGCCTA-3', CXCR4 forward primer, 5'-AAGGCTATCAGAAGCGCAAG-3', CXCR4 reverse primer, 5'-GAGTCGATGCTGATCCCAAT-3'. Amplicon size was between 100 and 150 base pairs for each primer set. The PCR efficiency for primer sets was assessed using cDNA standards, and each amplified at 95-110% efficiency over a broad (10,000 fold change in cDNA concentration) target range. The qPCR conditions were 95°C for 3 min followed by 40 cycles of 95°C (30 sec), 55°C (30 sec), 72°C (15 sec) and then a melt curve was performed per manufacture's conditions. All primer sets produce only one product, and the amplicon produced by each primer pair was sequenced to ensure that each gene of interest was correctly amplified. GAPDH expression did not change due to pregnancy status or gestational age and was used to normalize each target mRNA by using the ΔCt (target Ct - GAPDH Ct) values [19]. Data are represented by graphing 2^-ΔCt ^values calculated for each gene of interest.

### Western blot analysis

Protein lysates were collected from ovine tissues as described above. Equal concentrations of protein (20 μg per well for endometrium and intercaruncle and 50 μg per well for caruncle) were separated by SDS-PAGE using 10% polyacrylamide gels followed by transfer to nitrocellulose membrane for immunoblotting. Samples were analyzed for CXCR4 by Western blot analysis using an anti-CXCR4 antibody (sc-9046, Santa Cruz Biotechnology, Inc. Santa Cruz, CA) at 1:1000 dilution in 5% non-fat milk made in Tris-buffered saline plus tween (TBST). A secondary goat anti-rabbit IgG-horseradish peroxidase antibody (Santa Cruz Biotechnology, Inc. Santa Cruz, CA) at a dilution of 1:5000 was used for all Western blots. Proteins were detected using the Amersham ECL Plus Western Blotting Detection System (Amersham Pharmacia Biotech). Samples from ewes for each tissue analyzed (endometrium, caruncle, and intercaruncle), were subjected to electrophoresis on a single polyacrylamide gel and transferred to nitrocellulose. ECL signals were detected with a Storm imager and quantified with ImageQuant TL software (Amersham Pharmacia Biotech). An anti-actin antibody (sc-47778; Santa Cruz Biotechnology) was used at 1:1000 dilution for all Western blots to further demonstrate equal loading.

### Statistical analysis

Significant changes for the qPCR data were determined at *P *< 0.05 using an unpaired, two-tailed Student's *t*-test on normalized Ct values. Signals on western blots were scanned with a Storm imager and significant changes were determined at *P *< 0.05 using an unpaired, two-tailed Student's *t*-test.

## Results

**Figure 1:**
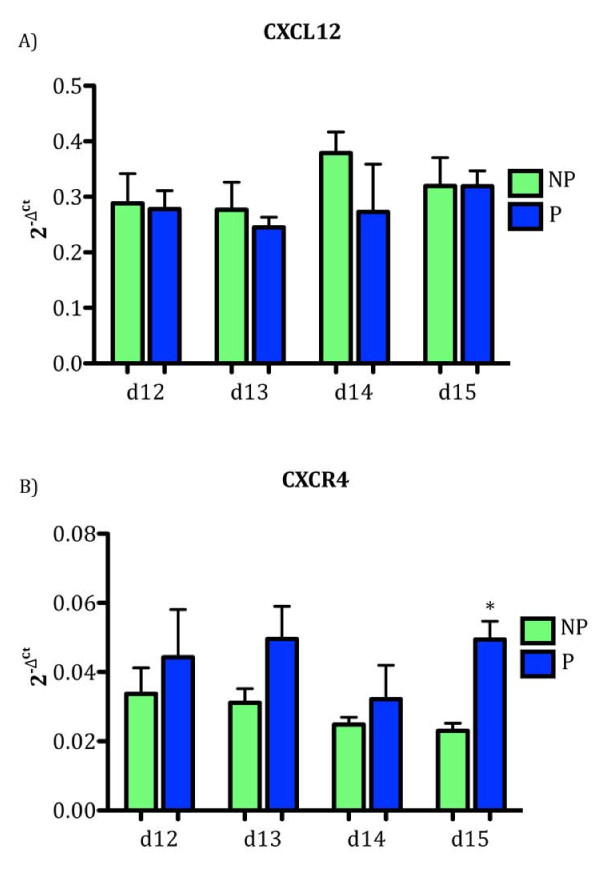
**Expression of mRNA for CXCR4 is greater in the endometrium on Day 15 of pregnancy (P) compared to Day 15 of the estrous cycle (NP), whereas CXCL12 mRNA concentrations did not change**. CXCL12 (A) endometrial mRNA did not differ in ewes on Days 12-15 of the estrous cycle or pregnancy. CXCR4 (B) mRNA was significantly (P < 0.05) increased on Day 15 of pregnancy compared to Day 15 of the estrous cycle. Data are represented by graphing 2^-ΔCt ^values, where ΔCt indicates *GAPDH *normalized Ct values. Data represent the mean ± SEM.

**Figure 2:**
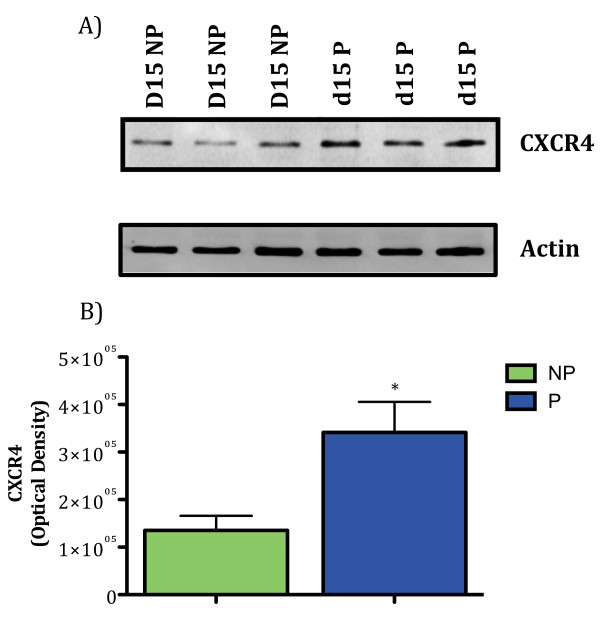
**CXCR4 protein concentrations are greater in the endometrium on Day 15 of pregnancy (P) compared to Day 15 of the estrous cycle (NP)**. Equal concentrations of endometrial protein from Day 15 pregnant and non-pregnant ewes were subjected to SDS-PAGE and Western blot analysis was performed to verify expression of CXCR4 (A). Data represent a minimum of 3 ewes per pregnancy status per day. The same protein samples were also immunodetected for actin to further verify equal loading of protein. Signals on Western blots were scanned on a STORM imager, converted to densitometric values, and analyzed. Values represent the mean ± SEM. Significant differences (P < 0.05) between endometrium from NP and P ewes are indicated by an asterisk (B).

CXCL12 mRNA was detected in the endometrium on all days tested but did not differ between pregnant and non-pregnant ewes (Figure 1A). CXCR4 mRNA was greater (*P *< 0.05) in the endometrium from pregnant compared to non-pregnant ewes on Day 15 of gestation (Figure 1B). Consistent with the increase in CXCR4 mRNA, an increase in CXCR4 protein production in the endometrium from pregnant ewes on Day 15 of gestation was confirmed by Western blot analysis (Figure 2).

**Figure 3:**
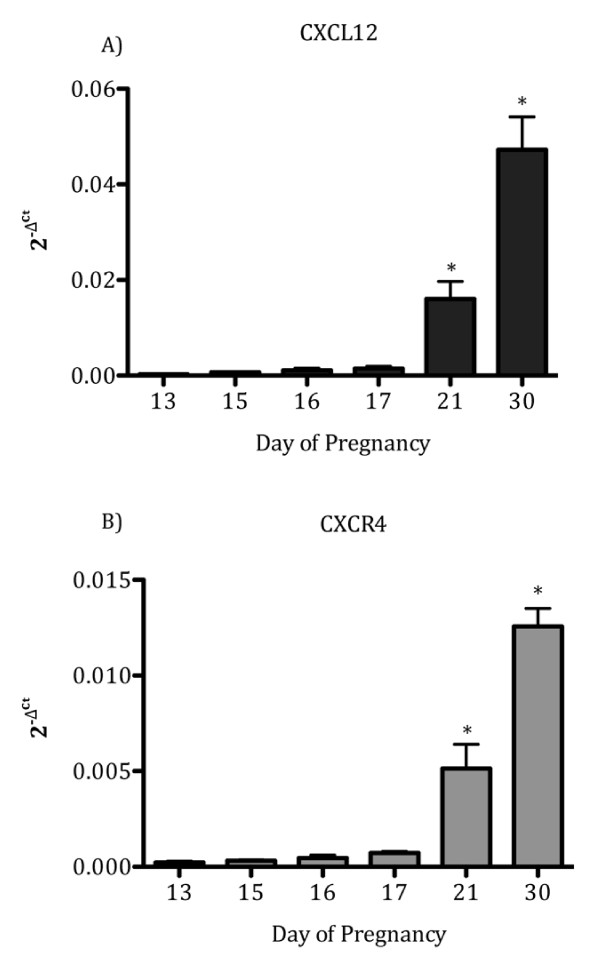
**CXCL12 and CXCR4 mRNA is upregulated in conceptuses during early gestation**. CXCL12 (A) and CXCR4 (B) mRNA are significantly (P < 0.05) upregulated in ovine conceptuses on Day 21 compared to earlier days collected. Both CXCL12 and CXCR4 transcripts are also significantly (P < 0.05) upregulated on Day 30 of pregnancy compared to Days 13, 15, 16, 17, and 21. Data are represented by graphing 2^-ΔCt ^values, where ΔCt indicates *GAPDH *normalized Ct values. Data represent the mean ± SEM.

To determine if gene expression for CXCL12 and CXCR4 changes in conceptuses during early gestation, qPCR was performed. CXCL12 and CXCR4 mRNA in ovine conceptuses exhibited a similar expression pattern across days tested increasing significantly (*P *< 0.05) in a time-dependent fashion by Day 21 of gestation, with the greatest expression noted on Day 30 (Figure 3).

**Figure 4:**
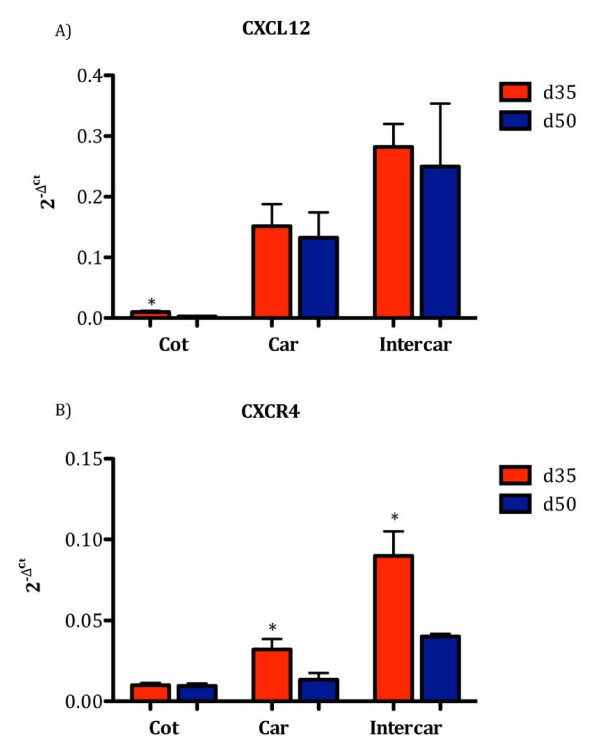
**Upregulation of mRNA for CXCL12 and CXCR4 on Day 35 compared to Day 50 of pregnancy in cotyledon (Cot), caruncle (Car) and intercaruncle (Intercar) tissue**. CXCL12 (A) was significantly (P < 0.05) greater in cotyledon tissue on Day 35 compared to Day 50 of gestation. CXCR4 (B) was significantly (P < 0.05) elevated in caruncle and intercaruncle tissue on Day 35 compared to Day 50 of pregnancy. Data represent 2^-ΔCt ^values, where ΔCt indicates *GAPDH *normalized Ct values. Data represent the mean ± SEM.

**Figure 5:**
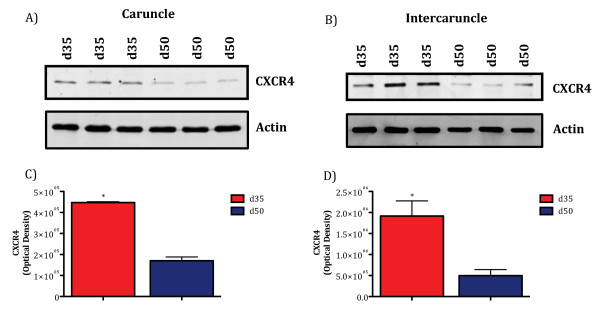
**CXCR4 protein concentrations are greater in caruncle and intercaruncle tissue on Day 35 compared to Day 50 of pregnancy**. Equal concentrations of caruncle and intercaruncle protein lysate from Days 35 and 50 of gestation were subjected to SDS-PAGE and Western blot analysis was performed to verify expression of CXCR4. Data are representative blots of a minimum of 3 ewes per day. CXCR4 was greater in caruncle (A) and intercaruncle (B) tissues on Day 35 compared to Day 50 of gestation. The same protein samples were also probed for actin to further verify equal loading of protein. Signals on Western blots were scanned on a STORM imager, converted to densitometric values, and analyzed. Values represent the mean ± SEM. Significant differences (P < 0.05) in caruncle (C) and intercaruncle tissues (D) from ewes on Day 35 and 50 are indicated by an asterisk.

To assess specific changes at the maternal/fetal interface, cotyledon, caruncle, and intercaruncle tissues were evaluated for CXCL12 and CXCR4. CXCL12 mRNA was greater (*P *< 0.05) in cotyledons on Day 35 compared to Day 50 (Figure 4A), whereas CXCR4 did not differ in cotyledon tissue between these two gestational ages (Figure 4B). On Day 35 of gestation, mRNA for CXCR4 was greater compared to Day 50 in both caruncle and intercaruncular tissue (Figure 4B), while mRNA for CXCL12 did not differ in these tissues between the two gestational ages (Figure 4A). To determine if changes in CXCR4 mRNA correlated with translation, Western blot analysis was performed using caruncle and intercaruncle tissue. Consistent with mRNA expression patterns, CXCR4 protein also was significantly greater (*P *< 0.05) in caruncle and intercaruncle tissue on Day 35 compared to Day 50 of gestation (Figure 5).

**Figure 6:**
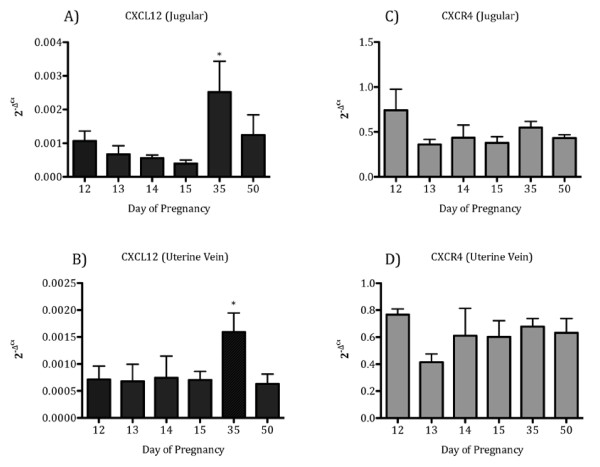
**CXCL12 mRNA is greater in white blood cells from jugular and uterine vein samples during pregnancy, while CXCR4 mRNA did not change**. CXCL12 (A) was significantly (P < 0.05) elevated in white blood cells from the jugular vein on Day 35 compared to Days 12, 13, 14, or 15 of gestation. CXCL12 (B) in white blood cells from uterine vein blood samples were also significantly (P < 0.05) elevated on Day 35 compared to Days 15 and 50 of pregnancy. CXCR4 (C, D) was detected in white blood cells collected on each day of pregnancy, but did not differ. Data are represented by graphing 2^-ΔCt ^values, where ΔCt indicates *GAPDH *normalized Ct values. Data represent the mean ± SEM.

CXCL12 mRNA in white blood cells from jugular and uterine vein blood samples was detected in non-pregnant and pregnant ewes on Days 12, 13, 14, and 15 but did not differ. However, on Day 35 of pregnancy, CXCL12 mRNA in white blood cells from jugular blood samples was greater (*P *< 0.05) compared to Days 13, 14 and 15 (Figure 6A). In uterine vein blood samples, mRNA for CXCL12 in white blood cells displayed a pattern similar to jugular vein blood with greater (*P *< 0.05) CXCL12 mRNA on Day 35 of gestation compared to Days 15 and 50 of gestation (Figure 6B). CXCR4 mRNA in white blood cells from jugular vein and uterine vein blood samples was detected on all days evaluated, but did not differ in pregnant or non-pregnant ewes. (Figure 6).

## Discussion

Unlike many other chemokine receptors, CXCR4 has only one recognized ligand, CXCL12 [20]. The CXCL12/CXCR4 system is implicated in cross talk between trophoblasts and endometrium, recruitment of lymphocytes into the uterus of pregnant females and vascularization. Based on known critical functions of CXCR4 activation during early pregnancy in other species, we hypothesized that CXCL12 and CXCR4 in the endometrium and conceptus increased in response to implantation and placentation in ewes to facilitate communication between trophoblast cells and maternal endometrium. To test this hypothesis, our first study investigated the expression of CXCL12 and CXCR4 in endometrium from pregnant and non-pregnant sheep. CXCR4 was consistently expressed in pregnant ewes across days tested, with decreasing CXCR4 in the endometrium from non-pregnant ewes with advancing days of the estrous cycle. On Day 15 of pregnancy we observed greater (*P *< 0.05) mRNA and protein for CXCR4 in the endometrium from pregnant compared to non-pregnant ewes. By Day 15 of gestation initial attachment of the conceptus to maternal endometrium begins in sheep [21]. As signal transduction pathways activated by the CXCL12/CXCR4 axis are known to regulate adhesion, as reviewed by Kucia and colleagues [22], it is plausible that CXCR4 may play a role during attachment. However, further study needs to be conducted to ascertain the role of CXCL12/CXCR4 signaling during implantation.

Coupled with initial attachment of the conceptus to maternal endometrium, recent studies reported by Grazul-Bilska and co-workers have demonstrated that angiogenesis is initiated very early in gestation as reflected by proliferation of capillary endothelial cells, as early as Day 16 of pregnancy in sheep [23]. The most potent inducer of both angiogenesis and microvascular permeability identified so far is vascular endothelial growth factor (VEGF). Gene-knockout (KO) studies have provided convincing evidence for a central role of VEGF in fetal and placental angiogenesis. A lack of even a single allele of VEGF or VEGFRs results in defective fetal and placental vasculogenesis and angiogenesis, culminating in embryonic death by mid-gestation [24-27]. Another potent angiogenic factor, basic fibroblast growth factor (bFGF, or FGF2), stimulates proliferation of both uterine arterial and fetal placental arterial endothelial cells [28-31] and is produced by both fetal and maternal placental tissues [32-35]. Thus, bFGF may function as an angiogenic factor during placentation. Interestingly, both VEGF and bFGF increase expression of CXCR4, but not other chemokine receptors [36]. Subcutaneous injection of CXCL12 into mice induces formation of local small blood vessels accompanied by leukocyte infiltration [37]. CXCL12 binding with CXCR4 on endothelial cells further amplifies angiogenesis by inducing more VEGF release [37,38]. VEGF and bFGF not only induce CXCR4, but also enhance CXCL12 production by endothelial cells [39], establishing a positive-feedback loop in which VEGF induces CXCR4 and CXCL12 expression, and conversely CXCL12/CXCR4 interactions enhance VEGF expression by these cells, consequently linking classic angiogenic factors to chemokine-induced angiogenesis. CXCL12/CXCR4 interactions induce expression of other angiogenic signals as well, specifically prostacyclin production by endothelial cells [40]. It should be emphasized that despite expression of multiple functional chemokine receptors on endothelial cells, only CXCL12/CXCR4 interactions are necessary for some, but not all types of angiogenesis [36]. Further, only CXCL12 and CXCR4 KO mice exhibit vascular abnormalities compared to other chemokines and their receptors [41]. Defects in vascularization have not been observed in any other chemokine receptor or ligand KO mice, thus their angiogenic role is, if anything, redundant.

The above provides strong support for CXCL12/CXCR4 interactions during vascularization and it is conceivable similar functions exist during placental development, which is characterized by extensive vascularization. In the present study CXCR4 increases between Days 15 and 35 of pregnancy with peak expression noted on Day 35 in maternal endometrium and subsequently decreasing by Day 50 of gestation. Interestingly, CXCL12 mRNA in white blood cells from both jugular and uterine vein samples exhibited a similar expression pattern, with peak CXCL12 observed on Day 35 of pregnancy when the greatest expression of CXCR4 is observed in the endometrium. The combined data are curious with respect to the timeframe of early pregnancy in sheep as the process of attachment and placentation is a prolonged process with initial attachment beginning on Day 15-16 of gestation, endometrial vascularization increasing on Days 20-22 and placentation not complete until Days 50-60 of pregnancy [23,42-44]. In our study, peak expression of CXCR4 in caruncle and intercaruncle tissue is observed on Day 35 compared to Day 50, when placentation should be close to completion. Given the strong role CXCR4 plays in vascularization in other systems, it is probable that activation of CXCR4 is also driving similar functions during placentation.

The present study demonstrated that mRNA for CXCL12 and CXCR4 in ovine conceptuses is increased from Day 17-21, which is intriguing as this time frame correlates with apposition of trophoblast cells with luminal epithelium of the endometrium where adhesion complexes are formed by Day ~21-22 [21,45]. Also, from Day 21-30 of gestation, during placentation, a dramatic increase in trophoblast CXCR4 and CXCL12 is noted in conjunction with the increased CXCR4 (mRNA and protein) in endometrium of pregnant ewes from Day 15-35. Literature with respect to CXCL12 and CXCR4 in ruminants is limited, but in women, activation of CXCR4 causes recruitment of lymphocytes into decidua and stimulates trophoblast proliferation and invasion [14-16]. Trophoblasts secrete CXCL12, which binds to CXCR4 on decidual cells resulting in increased activity of matrix metalloprotease (MMP) 9 and MMP2 [46]. MMP9 and MMP2 are critical determinants for trophoblast migration and invasion [47,48]. Wu and co-workers [16] demonstrated increased viability of trophoblast cells and activation of MAPK ERK1/2 pathway in trophoblast cells treated with recombinant CXCL12, suggesting that CXCL12/CXCR4 interactions play an important role in early pregnancy. In support of these findings, Jaleel and co-workers [49] showed CXCL12/CXCR4 signaling suppressed apoptosis and enhanced trophoblast survival through the MAPK pathway and speculated that a lack of CXCL12/CXCR4 signaling may result in significant utero-placental pathology. Activation of CXCR4 by CXCL12 also promotes CD4^+ ^T-cell survival and increases expression of cell survival genes while inactivating pro-apoptosis genes such as Bcl2 [50]. In pregnant mice, CXCL12 causes migration of CXCR4^+ ^T_reg _cells into the uterus and prevents embryo loss [51]. Therefore, the CXCL12/CXCR4 system may affect migration of immunocompetent cells into the uterus and aid in establishment and maintenance of maternal tolerance to the fetal allograft. In support of this in the ovine model, the percentage of endometrial CD45R^+ ^lymphocytes is greater in early pregnant ewes compared to cyclic ewes [52,53], but whether CXCL12/CXCR4 activation is functioning to drive this migration in sheep is not known. To date, no research has been conducted on CXCL12/CXCR4 interactions in placenta during early pregnancy in sheep. We interpreted the data from the current study to mean the CXCL12/CXCR4 pathway is activated during implantation and placentation in sheep and is likely playing a role in the communication between trophoblast cells and the maternal endometrium.

In summary, we have described upregulation of a unique chemokine, CXCL12 and its receptor, CXCR4 during early pregnancy in sheep in both fetal and maternal tissues. Historically, study of chemokines and their respective receptors have focused specifically on immune functions, yet research into their biological roles in other systems has grown extending the functional implications of chemokine receptor activation. The unique relationship between VEGF, bFGF and the CXCL12/CXCR4 system may provide novel insights into the temporal changes in expression of select angiogenic factors and their functions during placentation. In ruminants, interferon tau (IFNτ) is produced by trophoblast cells and serves as the classic signal for maternal recognition of pregnancy. Pertinent to the current study, CXCL12 was recently identified as a new IFNτ induced gene in cattle [54]. Whether similar gene regulation is observed in sheep is not known, but underscores the communication that exists between trophoblast cells and the maternal endometrium during early gestation with regards to the CXCL12/CXCR4 system. In conclusion, data from the current study provides a solid foundation for future study of CXCL12 and CXCR4 functions during implantation and placentation, specifically vascularization of the placenta and highlights the novel roles CXCL12/CXCR4 signaling may have during early pregnancy.

## Competing interests

The authors declare that they have no competing interests.

## Authors' contributions

RLA and AQA completed the experiments. RVA provided the conceptus tissues and TRH provided reagents and materials. RLA performed all the qPCR and Western blot studies and drafted the manuscript. All authors read and approved the manuscript.
